# A Systematic Review of the National Breast Implant Registry for Application in Korea: Can We Predict “Unpredictable” Complications?

**DOI:** 10.3390/medicina56080370

**Published:** 2020-07-23

**Authors:** Woo Jin Song, Sang Gue Kang, Bommie Florence Seo, Nam-Kyong Choi, Jung Ho Lee

**Affiliations:** 1 Department of Plastic and Reconstructive Surgery, Soonchunhyang University College of Medicine, Seoul 04401, Korea; pswjsong@gmail.com (W.J.S.); sgkang@schmc.ac.kr (S.G.K.); 2 Korean Academic Association of Aesthetic and Reconstructive Breast Surgery, Seoul 04146, Korea; bommiefseo@gmail.com; 3 Department of Plastic and Reconstructive Surgery, Uijeongbu St. Mary’s Hospital, College of Medicine, The Catholic University of Korea, Uijeongbu 11765, Korea; 4 Department of Health Convergence, Ewha Womans University, Seoul 03760, Korea; namkyong.choi@gmail.com; 5 Department of Plastic and Reconstructive Surgery, Bucheon St. Mary’s Hospital, College of Medicine, The Catholic University of Korea, Bucheon 14647, Korea

**Keywords:** breast implants, medical device, registries

## Abstract

*Background and Objectives:* Since silicone breast implants were introduced to the market several decades ago, the safety of breast implants has remained controversial. Recently, several studies have explored breast implant-associated anaplastic large-cell lymphoma (BIA-ALCL) and breast implant illness (BII). Several countries have developed national breast implant registries to improve the safety and quality of breast implant surgery. We performed a systematic review of the current status of national breast implant registries and propose a pilot form of an appropriate breast implant registry model for Korea. *Materials and Methods:* The systematic review was conducted in accordance with the “preferred reporting items for systematic reviews and meta-analyses (PRISMA) pro forma”. PubMed and Google Scholar databases were searched to identify all articles containing information on national breast implant registries. We limited the search to articles written in the English language from 2010 to 2020. Articles were reviewed by two independent authors. *Results:* A total of 63 articles related to national breast implant registries, registry principles and national breast implant registry annual reports were identified. After reviewing the literature, 25 national breast implant registry-related articles were included in the full-text synthesis. Currently, four countries, The Netherlands, Australia, Sweden, and the UK, have breast implant registries with well-formed sources for big data. Overall, similarities in data points were detected for three categories: implant-related complications, operation details, and device information. However, there were differences for each registry in terms of governance, funding, and capture rate. *Conclusion:* After reviewing other countries’ experiences, tentative datasets for the Korean Breast Implant Registry (K-BIR) were developed. The K-BIR can improve the quality of breast implant surgery in Korea by providing datasets on overall processes and outcome measures with quality indicators and risk adjustment factors. This approach will register characteristics of patients and monitor breast implants, complications, and surgical procedures to improve the outcomes of breast implant surgery in Korea. In addition, it can be used as a track-and-trace system with automated notifications to patients in the event of a product recall or other safety concerns related to a specific type of implant.

*Background and Objectives:* Since silicone breast implants were introduced to the market several decades ago, the safety of breast implants has remained controversial. Recently, several studies have explored breast implant-associated anaplastic large-cell lymphoma (BIA-ALCL) and breast implant illness (BII). Several countries have developed national breast implant registries to improve the safety and quality of breast implant surgery. We performed a systematic review of the current status of national breast implant registries and propose a pilot form of an appropriate breast implant registry model for Korea. *Materials and Methods:* The systematic review was conducted in accordance with the “preferred reporting items for systematic reviews and meta-analyses (PRISMA) pro forma”. PubMed and Google Scholar databases were searched to identify all articles containing information on national breast implant registries. We limited the search to articles written in the English language from 2010 to 2020. Articles were reviewed by two independent authors. *Results:* A total of 63 articles related to national breast implant registries, registry principles and national breast implant registry annual reports were identified. After reviewing the literature, 25 national breast implant registry-related articles were included in the full-text synthesis. Currently, four countries, The Netherlands, Australia, Sweden, and the UK, have breast implant registries with well-formed sources for big data. Overall, similarities in data points were detected for three categories: implant-related complications, operation details, and device information. However, there were differences for each registry in terms of governance, funding, and capture rate. *Conclusion:* After reviewing other countries’ experiences, tentative datasets for the Korean Breast Implant Registry (K-BIR) were developed. The K-BIR can improve the quality of breast implant surgery in Korea by providing datasets on overall processes and outcome measures with quality indicators and risk adjustment factors. This approach will register characteristics of patients and monitor breast implants, complications, and surgical procedures to improve the outcomes of breast implant surgery in Korea. In addition, it can be used as a track-and-trace system with automated notifications to patients in the event of a product recall or other safety concerns related to a specific type of implant.

## 1. Introduction

Since the first silicone breast implant entered the US market in 1964 (Dow-Corning Corp., Midland, MI, USA), breast augmentation and reconstruction with silicone implants has increased dramatically. According to the International Society of Plastic Surgery (ISAPS) global survey, over 1.8 million breast augmentation surgeries were performed worldwide in 2018, and in South Korea, over 50,000 such surgeries were performed in 2015 [1,2]. However, because a breast implant is a foreign body with a large surface, the safety of breast implants has been questioned. In the past, there have been two important safety concerns. The first concern (Dow Corning) was raised in 1982, when a claim regarding the potential association between breast implants and autoimmune diseases was raised. The second concern (Poly Implant Prostheses, PIP) arose in 2010, when French surgeons reported abnormally high rates of product rupture, and subsequent manufacturing site inspections revealed that the company had used non-approved industrial grade silicone containing only 10% of the approved gel [3,4]. The Dow Corning and PIP cases highlighted the importance of comprehensive scientific data to assess and monitor the safety of breast implants [5].

Recently, concerns have been raised about breast implant-associated anaplastic large-cell lymphoma (BIA-ALCL). The first case of BIA-ALCL was reported by Keech and Creech in 1997 [6], and in 2011, the US Food and Drug Administration (FDA) identified a possible association between breast implants and ALCL. In 2016, the World Health Organization (WHO) recognized BIA-ALCL as an entity and emphasized the importance of surgical management of the disease [7]. As many studies showed that this rare disease entity was more closely associated with certain types of breast implants (Allergan BIOCELL textured implants), the FDA requested that the company voluntarily recall implants in 2019 [8]. After these cases were reported, patients who received implants became very concerned, and lawsuits against the company are ongoing.

South Korea has also recently had cases of BIA-ALCL. Last year, the first case of BIA-ALCL was reported, and two cases have been reported to date. To help identify and prevent these unpredictable complications and enhance patient safety, some countries have developed breast implant registries. Currently, Australia (Australian Breast Device Registry; ABDR, since 2015), The Netherlands (Dutch Breast Implant Registry; DBIR, since 2015), the United Kingdom (Breast and Cosmetic Implant Registry; BCIR, since 2016), the United States (National Breast Implant Registry; NBIR, since 2019), and Sweden (Swedish Breast Implant Registry; BRIMP, since 2014) have developed optimal models by designing a recognized and effective dataset form [3,9,10,11,12,13,14,15]. Recently, a systemic review article regarding breast implant registries was published [14]. However, it does not reflect recent data. For example, it reviewed the UK Breast Implant Registry (UKBIR) data before 2004 and the Danish Registry for Plastic Surgery of the Breast (DPB) data before 2007. In addition, it has not reviewed the details of Australian Breast Device Registry which has the largest cumulative number of registered implants. Here, we will review and analyze the current status of national breast implant registries and propose a pilot form of an appropriate registry model for Korea and, hence, build a monitoring system for the detection of adverse events in breast implants.

## 2. Materials and Methods

The systematic review was performed to identify all studies related to national breast implant registries and databases by following the “preferred reporting items for systematic reviews and meta-analyses (PRISMA) pro forma”. The PubMed (accessed 20 March 2020) and Google Scholar (accessed 20 March 2020) interfaces were used for a systematic literature search. We limited the search to articles written in the English language and published between 2010 and 2020 to include only the most recent papers. The PubMed search was performed using medical subject headings (MeSH) and keywords. The following keywords to be included in journal title were “Breast” AND “device” OR “implant” AND “registry” OR “database”. The Google Scholar search was performed using free terms. The search terms were “breast implant registry” and “breast device registry”. The key words were used as MeSH terms or as a free text appearing in the title or abstract. In addition, we consulted academic literature sources related to registry principles and the annual reports of each of the various national breast implant registries based on the data found on registry websites and Google search results. Due to the observational nature of the data and heterogeneity of the methodologies, it was not possible to perform conventional bias analysis such as through the use of funnel plots. Instead, the registry output differed slightly by country. For example, in the case of the Breast and Cosmetic Implant Registry (BCIR), the number of breast implants in each category of operation was not registered. Furthermore, in the case of the Swedish Breast Implant Registry (BRIMP), the number of procedures was not stated in the annual report. Our primary focus was on the nation, year established, governance, funding, data collection, registry participation, and registry output by subgroup analyses.

## 3. Results

**Figure 1:**
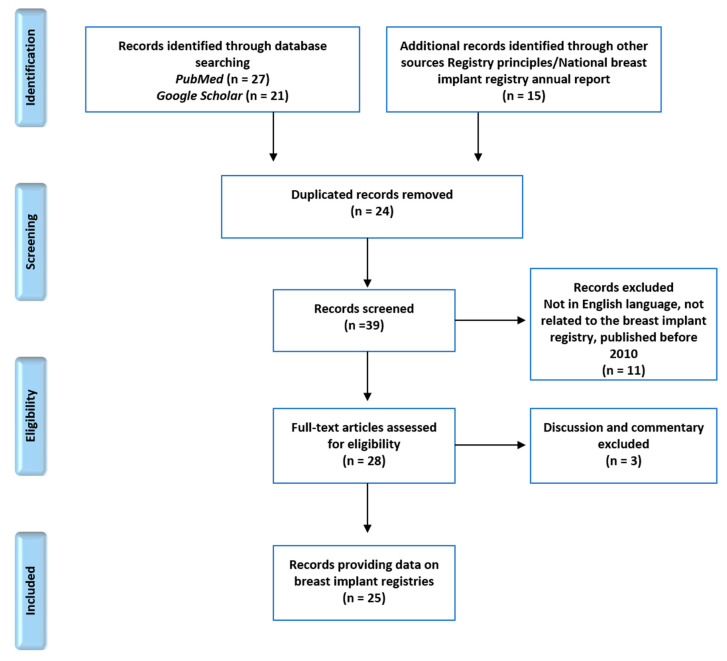
Preferred reporting items for systematic reviews and meta-analyses (PRISMA) diagram demonstrating the article selection process.

A total of 63 articles were reviewed. Forty-eight articles were identified from the two search interfaces using the previously mentioned search terms. Fifteen additional articles about registry principles and national breast implant registry annual reports from four countries were included in the review process. After removing 24 duplicates, 39 records were screened for national information on breast implant registries. Non-English articles, unrelated articles, and articles published before 2010 were excluded. In addition, articles containing only discussion or commentaries were removed. A total of 25 national breast implant registry-related articles were included in the full-text synthesis (Figure 1).

Currently, four countries have breast implant registries with well-formed sources for big data: The Netherlands (Dutch Breast Implant Registry; DBIR, since 2015), Australia (Australian Breast Device Registry; ABDR, since 2015), Sweden (Bröstimplantatregistret of Sweden; BRIMP, since 2014), and the UK (Breast and Cosmetic Implant Registry; BCIR, since 2016). A comparison of the four national breast implant registries is summarized in Table 1 [9,10,12,13]. Overall, similarities in data points were detected for three categories: implant-related complications, operation details, and device information. The US (National Breast Implant Registry; NBIR, since 2019) and Denmark (Danish Registry for Plastic Surgery of the Breast; DPB, since 1999) were excluded due to the start-up status and resultant short trial period and the lack of recent annual reports, respectively [16,17,18].

The DBIR register included characteristics of patients, procedures, and breast implants since April 2015. Recently, 96% of the hospitals and 69% of private clinics eligible for breast implant surgery participated in DBIR. From the start of the registry in April 2015 to the end of 2018, approximately 30,000 patients, 31,000 procedures, and 62,000 breast implants were registered. In 2018, approximately 3000 patients, 3500 procedures, and 4500 breast implants were registered for reconstructive indications, and 6300 patients, 6500 procedures, and 12,500 breast implants were registered for aesthetic indications. Overall, in 2018, the data capture rate was >90%. Registry output included indications for breast implant surgery, laterality, age, smoking, body mass index (BMI), intraoperative techniques (timing of reconstruction, incision site, plane, mastopexy, capsulectomy, autologous flap cover, fat grafting, drains, mesh/acellular dermal matrix use), infection control measures, revision surgery, and device characteristics (shape, texture, coating, fill, volume) [13,20]. With 2 full registration years, the data completeness of DBIR still needs to improve [21].

The ABDR commenced national rollout of data collection in June 2015. As of the end of 2018, the ABDR had collected data on 37,603 patients with 41,921 procedures involving 78,024 breast implants. Of all registered implants, 74% had a textured surface and 20.8% had a smooth surface in reconstructive indication, while 64.0% had a textured surface and 29.5% had a smooth surface in aesthetic indications. Capsular contracture was the most common complication in both reconstructive (39.6%) and aesthetic (40.9%) indication. The opt-out rate remained low, with only 1.1% of patients choosing to opt out of participating in the ABDR. In 2018, approximately 3300 patients, 3544 procedures, and 5200 breast implants were registered for reconstructive indications, and 8690 patients, 9337 procedures, and 18,000 breast implants were registered for aesthetic indications. Registry output included procedure numbers, procedure type, laterality, age, site type, intraoperative techniques (antibiotics, antiseptic rinse, glove change for insertion, antibiotic dipping solution, sleeve/funnel), device characteristics, (texture, shape, fill), acellular dermal/synthetic matrix use, complications, and revision incidence. Collection of patient-reported outcome measures (PROM) was rolled out nationally, showing a 78% response rate at 1-year follow-up in patients with reconstructive indications and 61% response rate with aesthetic indications [22]. Their work is being undertaken with the International Collaboration of Breast Registry Activity (ICOBRA) registries including The Netherlands, Sweden, UK, and US, towards a large combined annual report examining breast implants across these countries. This will ensure that when analyses of breast implants are undertaken in different countries, implants will be compared to other similar implants [9,23].

The BRIMP started enrolling patients in May 2014; approximately 15,000 patients and 25,000 breast implants have been registered with the aim to decrease complications and increase patient safety. In 2018, approximately 300 patients (400 breast implants) were registered for reconstructive indications, and 2600 patients (5100 breast implants) were registered with data for plane, incision site, and other surgical elements for aesthetic indications. The number of procedures was not stated in the annual report. The current total level of coverage of the BRIMP is approximately 65%. Registry output included indications for breast implant surgery; the number of patients; the number of breast implants; BMI; device characteristics (manufacturer, shape, texture); patient-reported data regarding dissatisfaction with breast prior to operation, plane, incision site, and other surgical elements; antibiotics; reoperations; and risk for reoperation. The BRIMP database reported data regarding the number of implants according to a manufacturer. Mentor had up about 55% (3018 implants) of reported implants during 2018, Motiva had 37% (2050 implants), while Allergan had 8% (424 implants). The number of smooth implants increased from 639 to 948 in 2018 due to increased awareness of BIA-ALCL. The BRIMP also has displayed robust data through the years regarding the age and BMI distribution. The age distribution in primary operation shows that 80% of patients were younger than 40 years old, and normal BMI distribution was seen in 80% of patients in all age categories. The BRIMP holds data from a total of 7694 number of breasts undergoing reoperations between 2014 to 2018 with 33% of breasts augmentations undergoing reoperation within 2 years of the index operation. The patients’ motivation for reoperation was mainly focused on desire for a change in breast volume or shape. The convergence of data from BRIMP with the ICOBRA data is planned [12].

The BCIR has been collecting data since the registry opened in October 2016. A total of 340 submitting organizations from England are currently registered to enter data in the registry. A total of 36,195 patients with 37,725 procedures were registered until June 2019. From July 2018 to June 2019, 2425 procedures were registered for reconstructive indications, and 9965 procedures were registered for aesthetic indications. The number of breast implants in each category of operation was not registered. Currently, the data suggest that the BCIR had an approximate case ascertainment of just over one-third. This suggests that nearly two-thirds of all people who had breast implant surgery in July 2017–June 2018 were not included in the registry. Registry output included the number of provider organizations, patients, operations, and breast implants; the category of operation; laterality, plane, incision site, other surgical elements; complications, intraoperative techniques (antibiotics, antiseptic rinse, glove change for insertion, antibiotic dipping solution, sleeve/funnel, nipple guards etc.), device, and mesh characteristics [10].

The results from four national breast implant registries showed that it can be used as an objective and transparent medical device evaluation system for post-marketing surveillance once the collected data have been effectuated.

## 4. Discussion

Compared with other implantable medical devices, breast implants have several unique characteristics. First, they are mainly used for improving quality of life for breast augmentation as well as reconstruction; i.e., patients can live without breast implants. For this reason, more accurate risk assessment is required, and tremendous anxiety or dissatisfaction is inevitable when unexpected complications occur. Second, although their use in breast reconstruction is covered by the National Health Insurance Service (NHIS) in South Korea, there is no NHIS benefit for breast augmentation. Hence, patients are reluctant to visit the hospital unless there are fatal complications from breast implants, and many complications can be under-recognized. In addition, early recognition of unexpected complications such as BIA-ALCL is not possible because patients cannot be informed of them preoperatively.

A medical device registry can provide safety systems for these conditions and has proven an integral part of the healthcare quality assurance system. By matching specific device data with patient data and recording surgical outcomes and complications, device safety or performance can be monitored, and early recognition of specific types of device failure becomes possible. In addition, with the breast implant registry, patients having high-risk implants can be tracked and informed of safety information when there are serious medical issues, such as BIA-ALCL or PIP fraud.

When building a breast implant registry (BIR), various approaches can be employed depending on the components of quality indicators (QIs) and risk adjustment factors (RAFs) selected [24]. The four national breast implant registries mentioned in the Results section have shown the greatest overlap in QIs and RAFs [9,10,12,13]. In the outcome category of QIs, various local (capsular contracture, device rupture, device deflation, device malposition, hematoma/seroma, infection) and systemic (BIA-ALCL, newly diagnosed breast cancer) complications are recorded. The QI process category includes operation details (laterality, operation type, incision site, drain use, antiseptic rinse, etc.), preoperative antibiotics, drains, and unique device identifiers (UDI; device manufacturer, device serial number, texture/shell, fill). RAFs include records of various patient risk factors (age, BMI, smoking, diabetes, chemotherapy, radiotherapy, acellular dermal matrix, or mesh etc.) that can affect surgical outcomes.

The more complex the dataset in the registry, the lower the surgeon participation, which affects data completeness and quality. Therefore, simplifying the dataset can reduce the time requirement and increase surgeon participation [25]. The ABDR and ICOBRA improved the quality of breast implant registry datasets by developing a minimum dataset form on a single page that can be completed in a few minutes to decrease complexity (Table 2) [26].

After reviewing other countries’ experiences with breast implant registries, tentative datasets for the Korean Breast Implant Registry (K-BIR) were developed by commission of the Korean Society of Plastic and Reconstructive Surgeons and the Ministry of Food and Drug Safety (MFDS). The final form was selected after an online survey of 14 active members of scientific committees in the Korean Academic Association of Aesthetic and Reconstructive Breast Surgery (Table 3 and Supplementary Material Figure S1). The resulting K-BIR pilot form includes patient demographic information, operation type, timing of reconstruction, device operation type, site details, elements of the operation, and revision details as QIs and medical history, smoking, and category of operation as RAFs. We collected the breast implant registry on a trial basis in consultation with the MFDS using this form and planned to adjust the QIs through feedback from various stakeholders in the future.

To maintain newly developed breast implant registries, several obstacles should be overcome. First, it is important to increase surgeon participation. To accomplish this, government organizations such as the MFDS must have strong initiatives and promote policies in a responsible manner, indicating that the obligation to patient safety lies not with the doctor who is the user of the medical device but with the government that regulates the medical device [25]. Completing and submitting the breast implant registry form remains complicated for doctors. It takes time and effort to obtain consent from patients and to enter and transmit data. Government agencies should secure a budget to operate the registry and establish an incentive system that benefits medical institutions participating in the registry. Financial incentives are known to be the most important factors motivating surgeon participation [27]. Although the MFDS does not currently have the authority to require registry participation, reimbursement, and coverage incentives through the government incentive program or from the manufacturer will encourage participation in the breast implant registry. Punishment or unreasonable incentive/disincentive systems should not be used, as they may lead to reports of false data.

Second, legislative support should be integrated, and an opt-out consent model should be mandatory for large population capture. An opt-in consent requires consent from surgeons and patients before the data can be included on a voluntary basis, whereas an opt-out consent allows surgeons and patients to enter into the registry at the time of the procedure and assumes consent unless they have indicated its withdrawal [28]. Typically, opt-in systems have a limited data capture rate ≤20% of all implants [29]. The opt-out model has proven effective in the national breast implant registries mentioned above, where the data capture rate reached approximately 70–90%, excluding the BCIR, which remains at 30% because entry into the BCIR was originally based on patient consent. However, the basis for collection has been changed from opt-in to opt-out, and consent for registry is no longer required for operations that took place from 14 January 2019; higher patient recruitment is anticipated [10].

Third, it is important to have a system in place to protect patients’ personal information [30]. People undergoing cosmetic or reconstructive breast surgery are sensitive to privacy and are often reluctant to disclose that they have had the surgery. Therefore, to increase patient participation in the breast implant registry, the security of patients’ personal information is extremely important, and there should be no risk of its being exposed. Furthermore, patients should be informed that if there is a problem with the safety of an implant in the future, these systems allow for faster detection and advanced warning.

**Figure 2:**
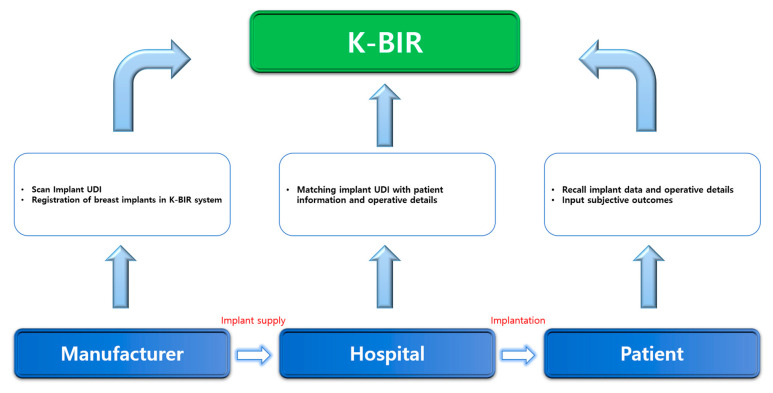
K-BIR system process.

Fourth, when gathering data, it is important to develop an interface suitable for producing statistics in a time-efficient manner. The current K-BIR pilot project will generate paper reports and transmit the data to MFDS. However, this is time consuming, and the accuracy may be low. Based on multiple consultations, we concluded that an electronic data entry system that can be used immediately in the operating theatre can facilitate data capture and potentially improve completion rates and data accuracy. Therefore, we propose an application platform that the manufacturer, surgeon, and patient can access. Within the system, it is possible to identify where the implant was used and for which patients. When the manufacturer produces the breast implant and delivers it to the medical institution, the corresponding UDI is scanned and registered by the application. As this implant with the UDI is inserted, operative details can be registered by the surgeon. Furthermore, when patients access the application, they can check the type, manufacturer, and surgical information of the breast implant through the individually linked UDI (Figure 2). Currently, South Korea has started the pilot study for national breast implant surgery, and about over 300 patients have been registered until now. Before registration, we have received informed consent from all patients. If the patient refuses to enroll, they are excluded from the registry. After registration, patients’ personal information undergoes a de-identification process and cannot be accessed. Their personal information can only be accessed when there is a newly developed safety issue, such as BIA-ALCL crisis, and it is necessary for patients to be informed of it. Manufacturers can only trace the status of implants they produced, whether they are used for patients or not. In other words, when implants remain in stock or are returned, their information is removed out from the registry and only implants used are left in the registry. The hospital has authority to access surgical and implant information of the patients they operated on, not all patients. Patients can be logged in the registry after identification with their resident registration number. The patient has the authority to access their own surgical and implant information, not that of other patients on the registry. After tripartite coordination, the K-BIR application can eventually become transparent with manufacturers, institutions, and patients. Moreover, PROM is not yet included in the tentative K-BIR dataset to increase the compliance rate, but the K-BIR application intends to include PROM in the future. Recently, the importance of subjective evaluation by patients, as well as the judgment of doctors, has been supported when determining surgical outcomes. In addition, although significant scientific evidence is not yet available, studies on breast implant illnesses are increasing [31,32,33,34,35]. Therefore, a platform must be developed in which the patient can evaluate the surgical outcomes on their own and obtain more information regarding the quality of all registered breast implants by linking patient feedback to the clinical data.

A breast implant registry can be helpful for post-market safety surveillance. Current sources of nationwide data for post-market surveillance rely on passively reported data that have been collected retrospectively after the occurrence of an adverse event. However, if we combine breast implant registry data with a unique computerized automated safety surveillance tool, such as the Data Extraction and Longitudinal Trend Analysis system (DELTA), the breast implant registry will provide prospective and continuous monitoring of adverse events related to breast implants [36].

The limitation of this study is that it is a comprehensive systematic review without meta-analysis. As the four national breast implant registries are currently providing active services with slightly different data collection, the details could not be compared. Finally, our research is limited to articles retrieved from PubMed and Google scholar.

## 5. Conclusions

We have analyzed eligible functioning national breast implant registries that are using similar datasets. Previously, Wurzer et al. (2019) published a systematic review of breast implant registries to summarize the published data based on the available registers. They reported three countries currently operating national breast implant registries (Austria, Australia, and United States). The systematic review analyzed time span, number of implants, and number of patients of the UKBIR, the DPB, the Austrian Breast Implant Register, and the DBIR. However, no detailed information or results from the UKBIR were shown since 2004, and the DPB has not published another follow-up article since 2007 [14]. There are some differences and novelties in our study when compared to this. We limited our search to only data published from 2010 to analyze the current trends in national breast implant registries. To the best of our knowledge, currently four countries are active in operating national breast implant registries with annual report (The Netherlands, Australia, Sweden, and the UK). We also analyzed what is included in the data collection form of each national breast implant registry and demonstrated the process of making the K-BIR based on this. The building of this the new breast implant registry is considered necessary on a national level. Integration of these datasets is important to compare and pool data from registries. The Korean Breast Implant Registry (K-BIR) can improve the quality of breast implant surgery in Korea by providing datasets on overall processes and outcome measures with QIs and RAFs. This approach will register characteristics of patients and monitor breast implants, complications, and surgical procedures to improve the outcomes of breast implant surgery in Korea. In addition, it can be used as a track-and-trace system with automated notifications to patients in the event of a product recall or other safety concerns related to a specific type of implant.

## Figures and Tables

**Table 1:** Analysis of the national breast implant registries [9,10,12,13,19].

	DBIR	ABDR	BRIMP	BCIR
**Since**	2015	2015	2014	2016
**Governance**	NVPC DICA	Monash University ACSQHC	Swedish Plastic Surgery Association and Swedish Society of Aesthetic Plastic Surgery	NHS Digital (Health and Social Care Information Center)
**Funding**	Financially covered by a fixed fee per implant (EUR 25).	Australian government	Financed through grants from the state and the regions. The Swedish plastic surgery association and the Swedish society for aesthetic plastic surgery have also provided financial support.	UK government
**Method of Enrollment**	Opt out	Opt out	Opt out	Opt out
**Data Capture Rate**	90% (2018) 88% (2017) 89% (2016)	74% (2018) 65% (2017) 44% (2016)	65%	33%
**Registry Output**	Patient demographics Device characteristics Case numbers (patient, implant, procedure) Intraoperative techniques Revision surgery Complications Infection control	Patient demographics Device characteristics Case numbers (patient, implant, procedure) Intraoperative techniques Acellular dermal matrix use Revision surgery Complications Infection control Breast implant-associated anaplastic large-cell lymphoma (BIA-ALCL) reports	Patient demographics Device characteristics Case numbers (patient, implant, procedure) Patient-reported data Infection control Revision surgery Risk for revision surgery	Patient demographics Device characteristics Case numbers (patient, implant, procedure) Intraoperative techniques Revision surgery Complications Infection control Mesh characteristics
**Cumulative Number of Registered Patients (Reconstructive/Aesthetic)**	8416/19,525	7870/28,090	1545/13,251	36,195 (Not classified)
**Cumulative Number of Registered Implants (Reconstructive/Aesthetic)**	17,722/42,919	16,542/57,952	1781/22,976	27,575 (Only reported from July 2018 to June 2019. Not classified by category of operation.)
**Number of Registered Patients in 2018 (Reconstructive/Aesthetic)**	3166/6550	3300/8690	295/2616	15,000 (Not classified)
**Number of Registered Implants in 2018 (Reconstructive/Aesthetic)**	6694/15,460	5218/17,815	391/5195	27,575 (Only reported from July 2018 to June 2019. Not classified by category of operation.)
**Registrations Per Year**	15,000–25,000	10,000–15,000	<5000	25,000–50,000

DBIR, Dutch Breast Implant Registry; ABDR, Australian Breast Device Registry; BRIMP, Bröstimplantatregistret of Sweden; BCIR, Breast and Cosmetic Implant Registry; ACSQHC, Australian Commission on Safety and Quality in Health Care; NVPC, The Netherlands Society of Plastic Surgery; DICA, Dutch Institute for Clinical Auditing, DICA; NHS, National Health Service.

**Table 2:** Breast device registry minimum dataset [26].

Breast Device Registry Minimum Dataset	
**Identifiers**	Patient demographics: Patient identifiers Device details: Device batch identifiers; manufacturer; and distributor Site details: Identifying physically separate operating theatres via name and address Surgeon details: Name of primary operating surgeon
**Additional Factors**	Patient history: Reason for primary operation; description of the operation; previous radiotherapy Elements of operation: Incision site; plane; mastopexy; use of mesh or acellular dermal matrix; use of fat grafting; tissue expander intraoperative fill volume Intraoperative techniques: Antiseptic rinse; antibiotic solution; prophylactic antibiotics; drains; sleeve/funnel (Keller funnel): nipple guards; glove change for insertion Revision operation: Description of operation; capsulectomy. Complications: Device rupture; device deflation; capsular contracture; silicone extravasation; device malposition; skin scarring problems; deep wound infection; seroma/hematoma; breast cancer; BIA-ALCL

BIA-ALCL, breast implant associated-anaplastic large cell lymphoma.

**Table 3:** Data items included in the K-BIR.

K-BIR Dataset (Pilot Form)		Category (QIs and RAFs)
**Patient Demographic**	Patient surname and first name Resident registration number Patient phone number	QIs (Structure)
**Patient History**	Medical history (diabetes, hypertension, breast cancer, other cancers)	RAFs
	Smoking	RAFs
	Category of operation (primary/revision surgery/insertion only/replacement/explantation only)	RAFs
	Operation type (cosmetic/reconstructive)	QIs (Process)
	Timing of reconstruction	QIs (Process)
	Device operation type (Implant/TE)	QIs (Process)
**Site Details**	Hospital name Business license number Surgeon name Department (plastic surgeon/general surgeon)	QIs (Structure)
**Device** **(Implant/TE/ADM)**	Manufacturer Device texture Device volume Lot number/serial number	QIs (Process)
**Elements of Operation**	Incision site (areolar/axillary/inframammary/mastectomy wound/mastopexy or reduction wound) Plane Laterality	QIs (Process)
**Revision Details**	Device rupture Device malposition Capsular contracture Breast implant illness Suspicious BIA-ALCL Seroma/hematoma Deep wound infection Palpable mass Related to patient’s desire Others	QIs (Outcome)

K-BIR, Korean Breast Implant Registry; TE, tissue expander; ADM, acellular dermal matrix; QIs, quality indicators; RAFs, risk adjustment factors.

## Supplementary Materials

The following are available online at https://www.mdpi.com/1010-660X/56/8/370/s1, Figure S1: K-BIR format.

## References

[1] ISAPS (2018). International Survey on Aesthetic/Cosmetic Procedures Performed in 2018. ISAPS Global Statistics.

[2] ISAPS (2015). International Survey on Aesthetic/Cosmetic Procedures Performed in 2015. ISAPS Global Statistics.

[3] Brown T., Merten S., Mosahebi A., Caddy C.M. (2016). Breast Implant Registries: The Problem with Ambition. Aesthet. Surg. J..

[4] Deva A.K., Cuss A., Magnusson M., Cooter R. (2019). The “Game of Implants”: A Perspective on the Crisis-Prone History of Breast Implants. Aesthet. Surg. J..

[5] Derby B.M., Codner M.A. (2015). Textured silicone breast implant use in primary augmentation: Core data update and review. Plast. Reconstr. Surg..

[6] Keech J.A., Creech B.J. (1997). Anaplastic T-cell lymphoma in proximity to a saline-filled breast implant. Plast. Reconstr. Surg..

[7] FDA/CDRH FDA Update on the Safety of Silicone Gel-Filled Breast Implants. https://www.fda.gov/media/80685/download.

[8] FDA The FDA Requests Allergan Voluntarily Recall Natrelle BIOCELL Textured Breast Implants and Tissue Expanders from the Market to Protect Patients: FDA Safety Communication. https://www.fda.gov/medical-devices/safety-communications/fda-requests-allergan-voluntarily-recall-natrelle-biocell-textured-breast-implants-and-tissue.

[9] Hopper I., Parker E., Pellegrini B., Mulvany C., Pase M., Ahern S., Earnest A., Cooter R., Elder E., Moore C. (2019). The Australian Breast Device Registry 2018 Annual Report.

[10] NHS Digital—Clinical Audit and Registries Management Service (2019). Breast and Cosmetic Implant Registry, BCIR Report 2019.

[11] Hopper I., Ahern S., Nguyen T.Q., Mulvany C., McNeil J.J., Deva A.K., Klein H., Stark B., Rakhorst H.A., Cooter R.D. (2018). Breast Implant Registries: A Call to Action. Aesthet. Surg. J..

[12] Stark B. (2018). BRIMP-Breast Implant Register Annual Report 2018.

[13] Becherer B.E. (2019). Dutch Breast Implant Registry (DBIR) Annual Report 2018.

[14] Wurzer P., Hundeshagen G., Cambiaso-Daniel J., Fischer S., Hoflehner H., Spendel S., Lumenta D.B., Kamolz L.P., Kneser U., Hirche C. (2019). Lessons Learned From Breast Implant Registries: A Systematic Review. Ann. Plast. Surg..

[15] Hopper I., Best R.L., McNeil J.J., Mulvany C.M., Moore C.C.M., Elder E., Pase M., Cooter R.D., Evans S.M. (2017). Pilot for the Australian Breast Device Registry (ABDR): A national opt-out clinical quality registry for breast device surgery. BMJ Open.

[16] Becherer B.E., Spronk P.E.R., Mureau M.A.M., Mulgrew S., Perks A.G.B., Stark B., Pusic A.L., Lumenta D.B., Hopper I., Cooter R.D. (2018). High risk device registries: Global value, costs, and sustainable funding. J. Plast. Reconstr. Aesthet. Surg..

[17] Henriksen T.F., Holmich L.R., Friis S., McLaughlin J.K., Fryzek J.P., Pernille Hoyer A., Kjoller K., Olsen J.H. (2003). The Danish Registry for Plastic Surgery of the Breast: Establishment of a nationwide registry for prospective follow-up, quality assessment, and investigation of breast surgery. Plast. Reconstr. Surg..

[18] Henriksen T.F., Holmich L.R., Fryzek J.P., Friis S., McLaughlin J.K., Hoyer A.P., Kjoller K., Olsen J.H. (2003). Incidence and severity of short-term complications after breast augmentation: Results from a nationwide breast implant registry. Ann. Plast. Surg..

[19] Rakhorst H.A., Mureau M.A.M., Cooter R.D., McNeil J., van Hooff M., van der Hulst R., Hommes J., Hoornweg M., Moojen-Zaal L., Liem P. (2017). The new opt-out Dutch National Breast Implant Registry—Lessons learnt from the road to implementation. J. Plast. Reconstr. Aesthet. Surg..

[20] Spronk P.E.R., Becherer B.E., Hommes J., Keuter X.H.A., Young-Afat D.A., Hoornweg M.J., Wouters M., Mureau M.A.M., Rakhorst H.A. (2019). How to improve patient safety and quality of care in breast implant surgery? First outcomes from the Dutch Breast Implant Registry (2015–2017). J. Plast. Reconstr. Aesthet. Surg..

[21] Becherer B.E., de Boer M., Spronk P.E.R., Bruggink A.H., de Boer J.P., van Leeuwen F.E., Mureau M.A.M., van der Hulst R., de Jong D., Rakhorst H.A. (2019). The Dutch Breast Implant Registry: Registration of Breast Implant-Associated Anaplastic Large Cell Lymphoma-A Proof of Concept. Plast. Reconstr. Surg..

[22] Ng S., Pusic A., Parker E., Vishwanath S., Cooter R.D., Elder E., Moore C., McNeil J., Hopper I. (2019). Patient-Reported Outcome Measures for Breast Implant Surgery: A Pilot Study. Aesthet. Surg. J..

[23] Ng S., Kirkman M., Fisher J., Pusic A., Parker E., Cooter R.D., Elder E., Moore C., McNeil J., Hopper I. (2019). Establishing the acceptability of a brief patient reported outcome measure and feasibility of implementing it in a breast device registry—A qualitative study. J. Patient Rep. Outcomes.

[24] Begum H., Vishwanath S., Merenda M., Tacey M., Dean N., Elder E., Mureau M., Bezic R., Carter P., Cooter R.D. (2019). Defining Quality Indicators for Breast Device Surgery: Using Registries for Global Benchmarking. Plast. Reconstr. Surg. Glob. Open.

[25] Wilkinson J., Crosbie A. (2016). A UK medical devices regulator’s perspective on registries. Biomed. Tech..

[26] Aloufi B., Alshagathrah F., Househ M. (2017). A Suggested Model for Building Robust Biomedical Implants Registries. Stud. Health Technol. Inform..

[27] Rahman S., Majumder M.A., Shaban S.F., Rahman N., Ahmed M., Abdulrahman K.B., D’Souza U.J. (2011). Physician participation in clinical research and trials: Issues and approaches. Adv. Med. Educ. Pract..

[28] Olver I.N. (2014). Opting in for opt-out consent. Med. J. Aust..

[29] Jeeves A.E., Cooter R.D. (2012). Transforming Australia’s Breast Implant Registry. Med. J. Aust..

[30] Ehrl P.A. (2019). On Implant-Registries. J. Epidemiol. Public Health Rev..

[31] Lachmansingh D.A. (2019). Breast implant illness and psychiatric implications. Ir. J. Psychol. Med..

[32] Magnusson M.R., Cooter R.D., Rakhorst H., McGuire P.A., Adams W.P., Deva A.K. (2019). Breast Implant Illness: A Way Forward. Plast. Reconstr. Surg..

[33] McGuire P.A., Haws M.J., Nahai F. (2019). Breast Implant Illness: How Can We Help?. Aesthet. Surg. J..

[34] Tang S.Y., Israel J.S., Afifi A.M. (2017). Breast Implant Illness: Symptoms, Patient Concerns, and the Power of Social Media. Plast. Reconstr. Surg..

[35] Rohrich R.J., Kaplan J., Dayan E. (2019). Silicone Implant Illness: Science versus Myth?. Plast. Reconstr. Surg..

[36] Vidi V.D., Matheny M.E., Donnelly S., Resnic F.S. (2011). An evaluation of a distributed medical device safety surveillance system: The DELTA network study. Contemp. Clin. Trials.

