# Supplementary material for: A Systematic Review of the National Breast Implant Registry for Application in Korea: Can We Predict “Unpredictable” Complications?
Source: Medicina (Kaunas). 2020 Jul 23;56(8):370. doi: 10.3390/medicina56080370 (PMC7466367; doi:10.3390/medicina56080370)
Supplement: Supplementary file 1 [file medicina-56-00370-s001.pdf]

Patient demographic	Name	
	Resident registration number	_ _ _ _ _ _ - _ * * * * * *
	Phone	
	Medical history	☐ Diabetes ☐ Hypertension ☐ Hepatitis ☐ Breast cancer ☐ Other cancers
	Smoking	☐ Current smoker ☐ Ex-smoker ☐ Nonsmoker
Informed consent	☐ Agreed	

Hospital information	Hospital name	
	Business number	
	Surgeon name	
	Department	

Surgical characteristics		Operation date	_ _ _ _ / _ _ / _ _	
Laterality	☐ Left ☐ Right<br>☐ Both	Number of operation	☐ Primary ☐ Secondary ☐ Tertiary	
			☐ More than quaternary (reason):	
Surgery type	☐ Tissue expander	☐ Partial/Total mastectomy	☐ Immediate	
			☐ Delayed	
		☐ Other:		
	☐ Implant	☐ Partial/Total mastectomy	☐ Immediate (DTI)	
			☐ Delayed	
			(with tissue expander removal)	
		☐ Augmentation mammaplasty		
		☐ Other:		
	☐ Tissue expander/ implant removal only			

Device characteristics	Manufacturer	
	Type of surface	
	Size	
	LOT No.	
	Serial No.	

Acellular dermal matrix information	Manufacturer	
	LOT No.	
	Serial No.	

Incision site	☐ Areolar
	☐ Axillary
	☐ Inframammary
	☐ Mastectomy scar
	☐ Mastopexy/Reduction scar
	☐ Other:

Explantation	☐ Device rupture	☐ Device malposition
	☐ Capsular contracture	☐ Breast Implant Illness
	☐ ALCL	☐ for ALCL diagnosis
	☐ Hematoma	☐ Seroma
	☐ Deep wound infection	☐ Mass
	☐ Patient wanted	☐ Other
